# Misdetection of frameshifts in SARS-CoV-2 genomes: need for additional harmonisation and efficient monitoring of data workflows

**DOI:** 10.1093/bioinformatics/btaf516

**Published:** 2025-09-15

**Authors:** Rok Kogoj, Mauro Petrillo, Samo Zakotnik, Alen Suljič, Miša Korva, Gabriele Leoni

**Affiliations:** Institute of Microbiology and Immunology, Faculty of Medicine, University of Ljubljana, Ljubljana, Slovenia; Seidor Italy S.r.l., Milan 20129, Italy; Institute of Microbiology and Immunology, Faculty of Medicine, University of Ljubljana, Ljubljana, Slovenia; Institute of Microbiology and Immunology, Faculty of Medicine, University of Ljubljana, Ljubljana, Slovenia; Institute of Microbiology and Immunology, Faculty of Medicine, University of Ljubljana, Ljubljana, Slovenia; European Commission, Joint Research Centre (JRC), Ispra, Italy

## Abstract

Five years after the outbreak of the SARS-CoV-2 pandemic in 2020, diagnostic laboratories have moved from massive sequencing of thousands of samples to routine surveillance of SARS-CoV-2 cases, as with all other respiratory viruses. Surveillance remains of paramount importance to prevent a further SARS-CoV-2 surge, as the virus has been shown to mutate rapidly and can render available drugs and vaccines ineffective. During the pandemic, several bioinformatics pipelines and workflows have been developed to streamline analysis, shorten turnaround time and ensure reproducibility. As the number of samples decreases, laboratories are moving towards more flexible sequencing strategies and optimizing the cost per sample. However, workflow redesigns, even if individual steps have proven successful time and time again, can lead to challenges when changes in a bioinformatics pipeline are introduced (e.g. version updates, implementation of new features, etc.), a new combination of viral mutations emerge or a change in wet-lab procedures leads to unpredictable results. Here, we present a report of misidentified frameshift mutations in the consensus sequence of SARS-CoV-2, which led to an incorrect assumption of mutations in the spike and nucleocapsid viral proteins with the potential to affect PCR detection or even antigen testing. This investigation exemplifies the need for better awareness of the challenges that can occur even when using routinely applied protocols and analytical workflows and highlights the need for cooperation between experts of NGS, bioinformaticians and decision-makers towards more harmonized data workflows.

## 1 Introduction

Genomic surveillance continues to play an important role in the management of SARS-CoV-2 infections. This virus can evolve into new variants that spread faster, evading existing antibodies from vaccines or previous infections and rendering antiviral agents ineffective (Ferreira *et al*. 2021; Davies *et al*. 2021; Tegally *et al*. 2021; Voloch *et al*. 2021; Manjunath *et al*. 2022). Genomic monitoring includes established protocols for sequencing, analysing and sharing data in actionable time (Lo and Jamrozy, 2020). These data are required to adjust possible non-pharmaceutical measures such as travel restrictions and test screenings, to help physicians select the right monoclonal antibodies for the therapy of critically ill patients, and to update vaccines. The most widely used database for sharing SARS-CoV-2 genomic data is the Global Initiative on Sharing All Influenza Data (GISAID) (Shu and McCauley 2017). At the time of writing, 17,151,401 SARS-CoV-2 sequences were stored in the database (Accessed on 20 January 2025). During the pandemic, Next-Generation Sequencing (NGS) laboratories were inundated with an unprecedented number of samples and challenges and used data workflows and pipelines for processing such amounts of sequences still under development. There was a shortage of scientists with sufficient bioinformatics skills to verify SARS-CoV-2 sequence quality, and not enough time to check each sequence before release. Despite differences in sequences quality, rapid publication of SARS-CoV-2 genomes was encouraged due to the pandemic, with minimum requirements such as <50% N stretches. Unconfirmed frameshifts were labelled ‘Frameshift not confirmed’ in GISAID (https://gisaid.org/resources/statements-clarifications/data-quality-control-procedures-and-b1621-mu-monitoring/, accessed on 16 January 2025), a common practice currently still in place.

In September 2024, 197 sequences from Slovenia sparked particular interest, since 3 peculiar mutations were consistently observed in all of them. Two in the spike (S) protein gene coding region and one in the nucleocapsid (N) protein gene coding region. The mutations in the S gene were annotated as ins22036A/A22036G and del23009_23012, which together form a ‘create+recover’ frameshift. These mutations resulted in two distant open reading frames (ORFs), each encoding a portion of the S protein. The mutation in the N gene annotated del28368_28369 was predicted to produce a truncated N protein or (more likely) to fuse ORF9b to the N protein. The result would be a “hybrid” ORF9b-N protein with the first 25 amino acids of the ORF9b protein followed by the 25–419 (to the end) portion of the N protein, which would basically cause a depletion of ORF9b. Numerous mutations and frameshifts are readily observed in SARS-CoV-2 published genomes, but these caught attention since *in silico* analysis showed that they may potentially affect some PCR detection methods currently used for diagnostics purposes (Marchini *et al*. 2023). In addition, nonsynonymous mutations occurring in nucleocapsid protein-encoding gene could potentially affect rapid antigenic tests, which in large part detect the N protein (Hagag *et al*. 2022).

A deep investigation into both raw data and data workflows was triggered to shed more light on these mutations because, if confirmed, such a new SARS-CoV-2 sublineage would have a global impact on tools used for diagnostics and a possible biological advantage compared to other sublineages. Our results highlight potential challenges when following standard protocols and analytical workflows.

## 2 Reanalysis of sequencing data

**Figure 1.**
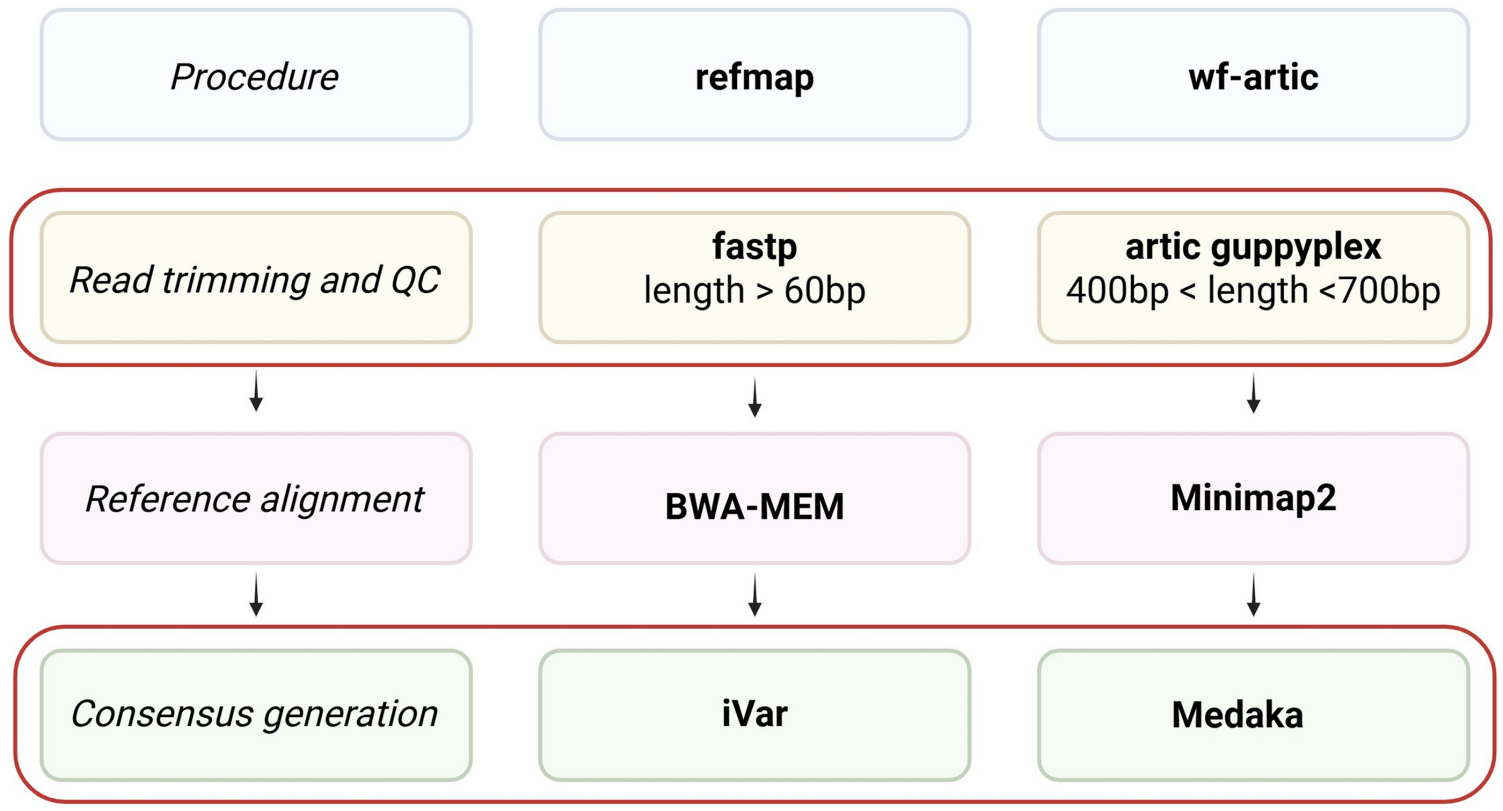
Step-by-step comparison of refmap and wf-artic bioinformatic pipelines. Circled boxes represent the steps where the discrepancies between the two might have been introduced

To assess the reliability of the identified mutations, we selected one pilot Slovenian sample (id: 8546) with all three mutations in question to be further analysed. This sample, like all others, was initially sequenced by Oxford Nanopore Technologies (ONT). Raw data analysis was performed with refmap, an established *in house*, EQA-controlled data analysis workflow in place in our lab from the beginning of the pandemic [https://github.com/NGS-bioinf/refmap] and for the purposes of this work, later also with wf-artic. A comparison between the two bioinformatic pipelines is shown in Figure 1, and the full description of materials and methods, with additional results details, is available as Supplementary Material.

ONT sequencing of the pilot sample generated 722,454 reads that, by using the refmap pipeline, amounted to 99.8% SARS-CoV-2 genome coverage with 8422× mean depth. From the same raw data, the wf-artic pipeline produced a consensus genome with a coverage of 99.3%.

Amplicons targeting SARS-CoV-2 regions of interest with the indicated mutations ins22036A/A22036G, del23009_23012 and del28368_28369 were successfully obtained (Supplementary Fig. S1, available as supplementary data at *Bioinformatics* online) and sequenced in both directions by Sanger sequencing. Trace files were merged into consensus sequences based on overlapping regions and used for further alignment analysis (Supplementary Fig. S2, available as supplementary data at *Bioinformatics* online).

From the consensus sequence alignment, ins22036A/A22036G turned out to be a *de facto* insA22034+G, regardless of sequencing technology (Sanger, ONT) or pipeline used (refmap, wf-artic). However, an unknown nucleotide (*n*) is additionally inserted at position 22032 in the ONT consensus sequence obtained with the refmap pipeline (Supplementary Fig. S3, available as supplementary data at *Bioinformatics* online). Detected ins_A22034+G has a coverage of 8188× and a frequency of 0.99.

In the second region of interest (del23009_23012), a deletion was detected with Sanger and NGS (Supplementary Fig. S3, available as supplementary data at *Bioinformatics* online). However, the size of the deletion is not identical. The consensus Sanger sequence and the wf-arctic pipeline obtained sequences show a three-nucleotide deletion (–TGT), while in the refmap pipeline obtained sequence, the deletion is four nucleotides long (–TGTT) followed by an unknown (n) nucleotide. A detailed review of NGS data revealed that the refmap pipeline recognizes several variants at the site of interest. The most prominent is the correct deletion del_G23007-TGT with a coverage depth of 13 158× and a frequency of detected deletion of 0.98. The second variant is T23008-GTTGA with a coverage depth of 186 and a frequency of 0.60. The third variant is T23008-GTTG with a coverage depth of 186 and a frequency of 0.27. Interestingly, the most frequent (correct) deletion was not carried through into the NGS consensus sequence.

At the N gene region of interest, del_G28368-CA was only located in the consensus sequence obtained with the refmap pipeline. All other analyses revealed a consensus sequence with a larger deletion del_G28361-GAGAACGCA (Supplementary Fig. S3, available as supplementary data at *Bioinformatics* online). The variant call of the pipeline refmap correctly recognizes the deletion del_G28361-GAGAACGCA with a coverage depth of 12 599× and a frequency of 0.98, which was, however, not retained in the consensus sequence.

Interestingly, by inspecting the aligned reads before refmap variant calling, ins22034+G and del_G23007-TGT can be readily observed as they should be in the consensus. However, del_G28368-CA is seen as the erroneous 9-nucleotide deletion (Supplementary Fig. S4, available as supplementary data at *Bioinformatics* online).

Based on the pilot sample observed, a discrepancy between refmap and wf-artic, further backed by Sanger sequencing, raw data of all 197 Slovenian sequences deposited in GISAID from July to September 2024 were retrieved from a local server and reanalysed by the wf-artic pipeline. All 197 sequences were found free of questionable mutations and resubmitted to GISAID as version 2.

## 3 Discussion

Our analysis shows how the combination of bioinformatics tools coupled with specific sequencing information can, in certain circumstances, generate hard to spot, differently annotated consensus sequences. This resulted in 197 sequences that had an apparent insertion and deletion in the S gene which together ‘create+recover’ a frameshift and a ‘hybrid’ ORF9b-N truncated protein. Both mutations caught special attention because *in silico* analysis showed potential impact on diagnostic PCR protocols and antigenic testing. At the time of uploading the initial sequences, the frameshifts were noticed but selected as confirmed based on the available bioinformatics metrics showing good genome coverage, high amounts of reads and very high sequencing depth. This first led to the assumption that a putatively new SARS-CoV-2 sublineage might have emerged; however, further NGS-independent, confirmation was deemed necessary. Therefore, targeted Sanger sequencing of the regions in question was performed, which ultimately disproved the presence of new mutations and frameshifts in the SARS-CoV-2 genome of the pilot sample. Consequently, an additional inspection of the refmap bioinformatics pipeline was triggered, as the mutations detected in the consensus sequences were found to be artefacts with Sanger sequencing. Refmap results were compared to the results obtained from the widely used pipeline wf-artic, which ultimately produced no such artefacts in our tests. Raw data from all 197 samples were then re-analysed, quality checked and uploaded to GISAID as version 2 of the published sequences. Thus, all sequences are now corrected and should not have any negative impact on further analysis or research. The identification of an additional 1072 SARS-CoV-2 sequences with the ins_A22034+G pattern (labeled as both ins22036A and A22036G) in the GISAID database may also be the result of annotation errors, similar to our findings, which would require further verifications. Moreover, by manually inspecting the alignment of reads to the reference sequence using refmap, both mutations in the S gene can be observed as correct, while the deletion in the N gene is seen as erroneous.

Visual inspection of read alignments to highlight false positive calls has been a key component of variant detection since the advent of NGS. For instance, in the oncology field, sequencing and alignment errors can be easily mistaken for somatic mutations, having a direct clinical impact (Robinson *et al*. 2017). However, this step came with a cost. It is time-consuming and requires highly skilled personnel trained to distinguish the patterns of true positive variants from false calls. The importance of training in the interpretation of sequence alignments and genome visualization has become increasingly evident, especially during global health crises such as the COVID-19 pandemic. The rapid expansion of genomic surveillance to fight the pandemic generated an unprecedented amount of sequencing data, which posed a significant challenge to traditional manual inspection procedures. The volume of sequences to be inspected, coupled with the urgent need for timely results, made it impractical to rely solely on manual inspection. As a result, there was a pressing need to adopt automated variant calling pipelines to quickly and accurately analyse the vast amounts of data being generated. This shift towards automation was driven by the necessity to keep pace with the rapidly evolving pandemic, where timely identification of variants was critical for informing public health decisions.

The pandemic also had a profound impact on training models, often limiting access to in-person instruction and mentorship. Initiatives like the Galaxy Training Network (GTN) (Hiltemann *et al*. 2023) have attempted to bridge this gap by offering cloud-based, hands-on tutorials for alignment and variant visualization using computational platforms like Galaxy (Galaxy Community, 2024) and federated cloud platforms such as AnVIL (Schatz *et al*. 2022). Similarly, the World Health Organization (WHO) highlighted the need for scalable training programs to equip laboratories with the skills necessary for genomic data interpretation and sharing (WHO 2022). Taken together, this shows the complexity and multilayer nature of the issue and underscores challenges associated with the training in the interpretation of sequence alignments and genome visualization, bioinformatics analysis of SARS-CoV-2 data, and more in general to any genomic NGS read set to be processed, as the final assembled sequences were produced using different sequencing techniques and platforms and analysed with different bioinformatics pipelines. The case presented in this article also stresses the importance and the need for methodological reference standards (e.g. benchmarking datasets and analyses, reference materials) and improved quality controls in the context of bioinformatics, when this is expected to generate trustworthy evidence in support of policymaking, such as in public health preparedness and response.

The bioinformatics processing of data consists of the application of a series of procedures. However, as our work evidently shows, some of these procedures, such as the trimming and variant calling step, entirely depend on the chosen bioinformatics software (and parameters), which can give different results if changed. A very well-document challenge in this context is the occurrence of frameshift errors in genome assemblies generated using long-read sequencing technologies, including ONT and Pacific Biosciences (PacBio) continuous long reads (CLR). To mitigate these issues, polishing steps have become essential, particularly when these technologies are adopted for genomic surveillance purposes. Tools like GoldPolish-Target have recently been developed to address this need by enabling targeted correction of indel and mismatch errors, achieving base-level accuracies exceeding 99.9% in both *Drosophila melanogaster* and *Homo sapiens* genome assemblies (Zhang *et al*. 2025). Similarly, polishCLR (Chang *et al*. 2023), a reproducible Nextflow workflow (Di Tommaso *et al*. 2017), implements best practices for polishing PacBio CLR assemblies and has demonstrated substantial improvements in assembly quality through multiple rounds of polishing. These developments underscore the importance of incorporating polishing steps in genome assembly pipelines to correct frameshift-inducing errors and enhance the reliability of genomic sequences to be shared.

Consequently, especially in a decisional context based on science evidence, the robustness of bioinformatics steps can be a critical point of weakness if not properly evaluated, as they influence reliability of key outcomes, for example, referring to the here presented SARS-CoV-2 case, the final sequence, the correct identification of mutations and the lineage definition and characterization. In the field of health, WHO recommendations on the generation and use of genomic data for surveillance purposes (https://www.who.int/initiatives/genomic-surveillance-strategy) indicate that genomics technologies and associated bioinformatics approaches have become key to contrast global health threats. Moreover, the adoption of these in support of policy-making is not confined to these threats. By 2030, the European Food Safety Authority (EFSA) is expected to routinely apply Omics Technologies (-omics) and bioinformatics in relevant risk assessments (RAs), to support the transition into a next generation food safety RA (Authority (EFSA) *et al*. 2022). Altogether, these examples underlie how -omics data, bioinformatics approaches and applications will surge within the policy context for One Health. These current and future applications of –omics and bioinformatics, in combination with the observation we made with our work reflex the strong need for additional harmonization and efficient monitoring of data workflows through agreed bioinformatics methodological reference standards to ensure better reproducibility, reduce the possibilities to introduce artefacts, produce more comparative sequences and provide high-quality and trustworthy results. We hope that our case will increase attention on this need, and will foster better cooperation between experts of NGS, bioinformaticians and decision-makers.

## Supplementary Material

btaf516_Supplementary_Data

## Data Availability

NGS and Sanger sequencing data that supports the findings regarding SARS-CoV-2 of this study are openly available in GISAID’s EpiCoV database: EPI_SET_250715cm at: https://doi.org/10.55876/gis8.250715cm. All 197 SARS-CoV-2 fasta sequences that have been corrected due to the findings of this study are openly available in GISAID’s EpiCoV database, EPI_SET_250715ec at: https://doi.org/10.55876/gis8.250715ec. Host-depleted fastq files are available at the European Nucleotide Archive (ENA): PRJEB96403. Host depletion was carried out using the tool *hostile* (Constantinides *et al*. 2023) with default parameters. We strongly recommend including a host depletion step before sharing raw data to ensure compliance with the EU data protection framework. The in-house Bioinformatic pipeline refmap is available at https://github.com/NGS-bioinf/refmap and at Zenodo: https://doi.org/10.5281/zenodo.15877026 Other data that support the findings of this study are available from the corresponding author upon reasonable request.
