# Supplementary material for: Misdetection of frameshifts in SARS-CoV-2 genomes: need for additional harmonisation and efficient monitoring of data workflows
Source: Bioinformatics. 2025 Sep 15;41(10):btaf516. doi: 10.1093/bioinformatics/btaf516 (PMC12557093; doi:10.1093/bioinformatics/btaf516)
Supplement: btaf516_Supplementary_Data [file btaf516_supplementary_data.pdf]

SUPPLEMENTARY MATERIAL

Contents

S.1 Amplification of SARS-CoV-2 genome regions harbouring investigated mutations for Sanger sequencing 2

S.2 Sanger sequences analysis of GISAID-Labelled Variants in SARS-CoV-2 Consensus Sequences 3

S.3 Alignment of SARS-CoV-2 consensus sequences obtained by Sanger Sequencing and ONT data analysed by two bioinformatic pipelines 4

S.4 Alignments of refmap obtained reads to the SARS-CoV-2 reference sequence 5

S.4 Methods 6

S.4.1 SARS-CoV-2 lineage monitoring program protocol 6

S.4.2 Targeted Sanger sequencing 6

S.4.3 Additional bioinformatic analysis 6

S.4.4 Re-analysis of all questionable sequences raw data 6

S.5 Supplementary tables 7

References 8

S.1 Amplification of SARS-CoV-2 genome regions harbouring investigated mutations for Sanger sequencing

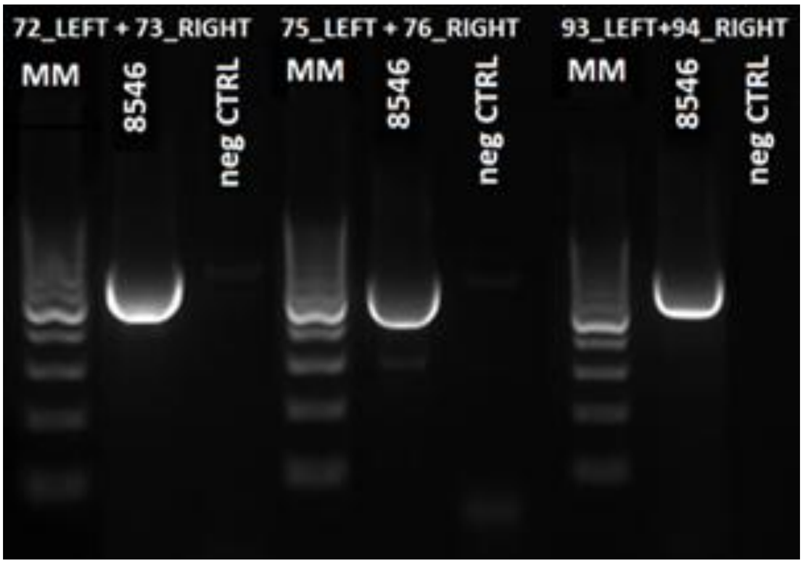

Supplementary Figure S1: Electrophoresis results of the pilot sample (8546) for all three tested SARS-CoV-2 genome regions intended for Sanger sequencing using appropriate primers from the ARTIC scheme (v.4.1). MM – molecular marker, 8546 - pilot sample, neg CTRL – negative control (water), 72_LEFT+73_RIGHT – primer pair for amplicon covering region: 21658 - 22324, 75_LEFT+76_RIGHT – primer pair for amplicon covering region 22516 – 23192, 93_LEFT+94_RIGHT - primer pair for amplicon covering region 28081 – 28756.

S.2 Sanger sequences analysis of GISAID-Labelled Variants in SARS-CoV-2 Consensus Sequences

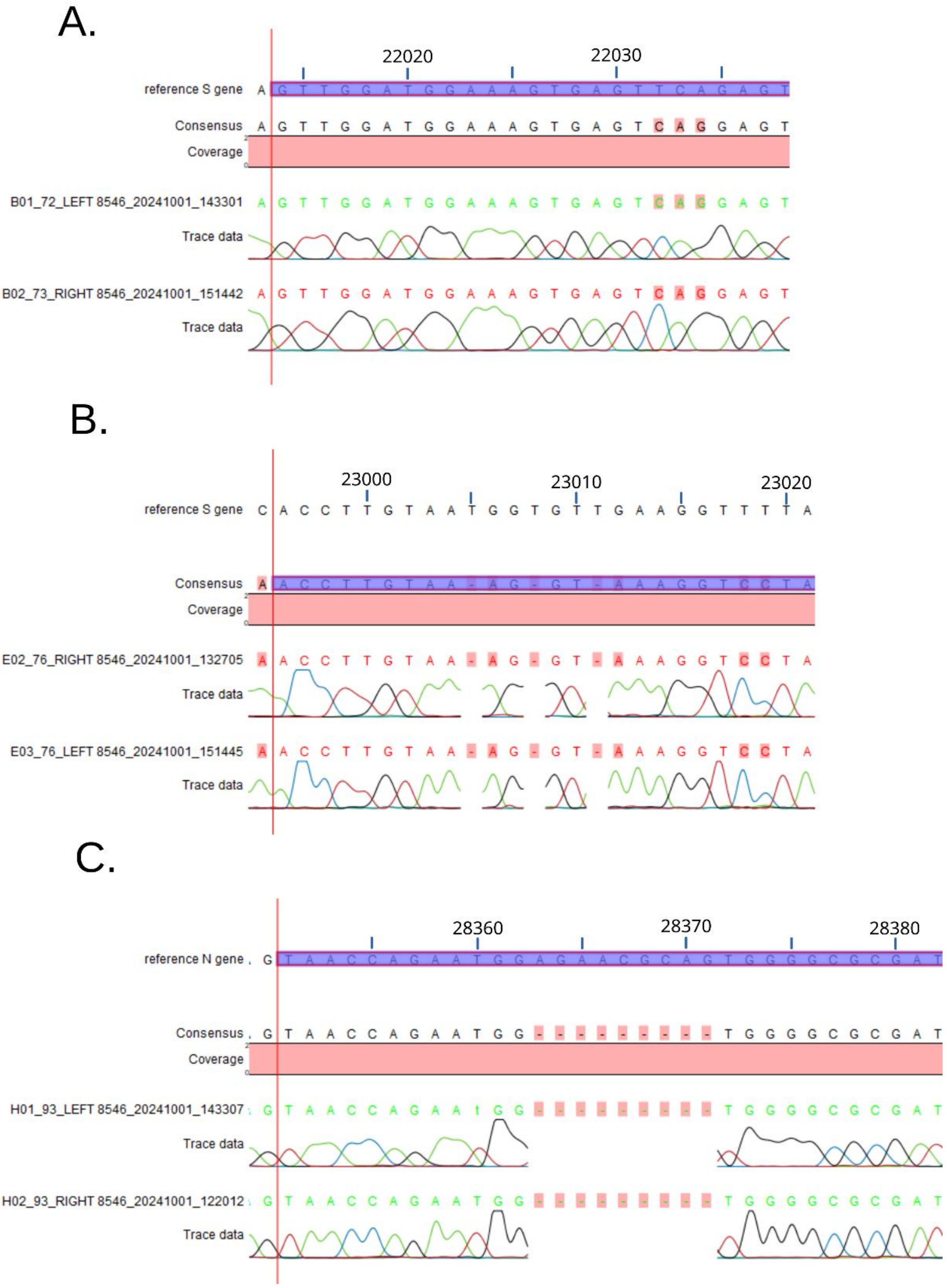

Supplementary Figure S2. Sanger sequences for the three regions of interest showing the alignment between obtained sequences for the pilot sample 8546 and appropriate parts of the reference Wuhan Hu-1 genome (NC_045512.2): **A.** Alignment of pilot sample 8546 Sanger trace files to S gene reference sequence in the region of interest (22014 – 22038), **B.** Alignment of pilot sample 8546 Sanger trace files to S gene reference sequence in the region of interest (22996 – 23021), **C.** Alignment of pilot sample 8546 Sanger trace files to N gene reference sequence in the region of interest (28351 – 28382).

S.3 Alignment of SARS-CoV-2 consensus sequences obtained by Sanger Sequencing and ONT data analysed by two bioinformatic pipelines

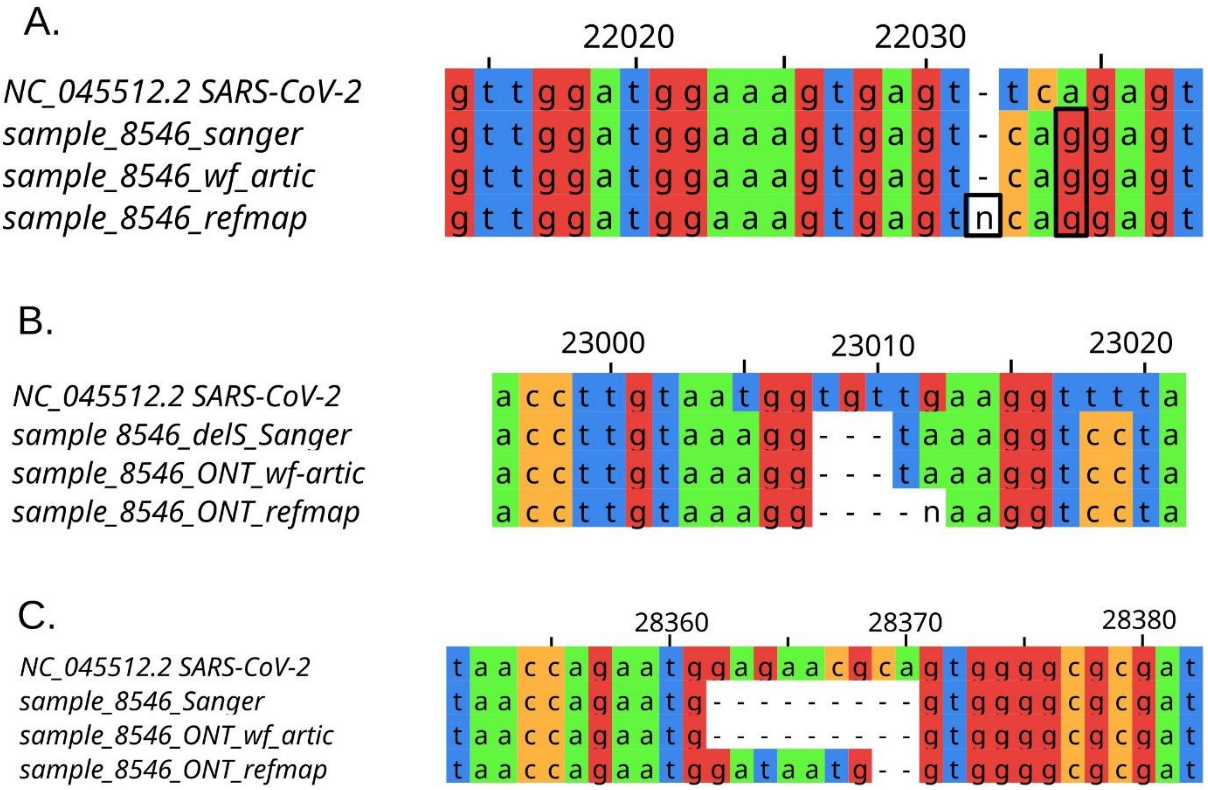

Supplementary Figure S3: Alignments of consensus sequences obtained by Sanger sequencing, ONT + wf-artic and ONT+ refmap at regions of interest **A.** Site of ins_A22034+G. Ins_A22034+G (black square) was detected and confirmed with both sequencing technologies and both analysis pipelines, however an anomaly (n) can be seen in ONT data processed by the refmap pipeline most probably accountable for the annotation ins22036A/A22036G in GISAID; **B.** Site of deletion del_23008- in the S gene of SARS-CoV-2. The deletion del_23008- was detected and confirmed with Sanger and both NGS sequencing technologies and both analysis pipelines but with a different length in the consensus genome. **C.** Site of deletion del_G28368-CA in the nucleocapsid protein of SARS-CoV-2. The deletion del_G28368-CA was found in the consensus sequence generated only with the pipeline refmap. All other consensus sequences have a longer, correct deletion of G28361-GAGAACGCA.

S.4 Alignments of refmap obtained reads to the SARS-CoV-2 reference sequence

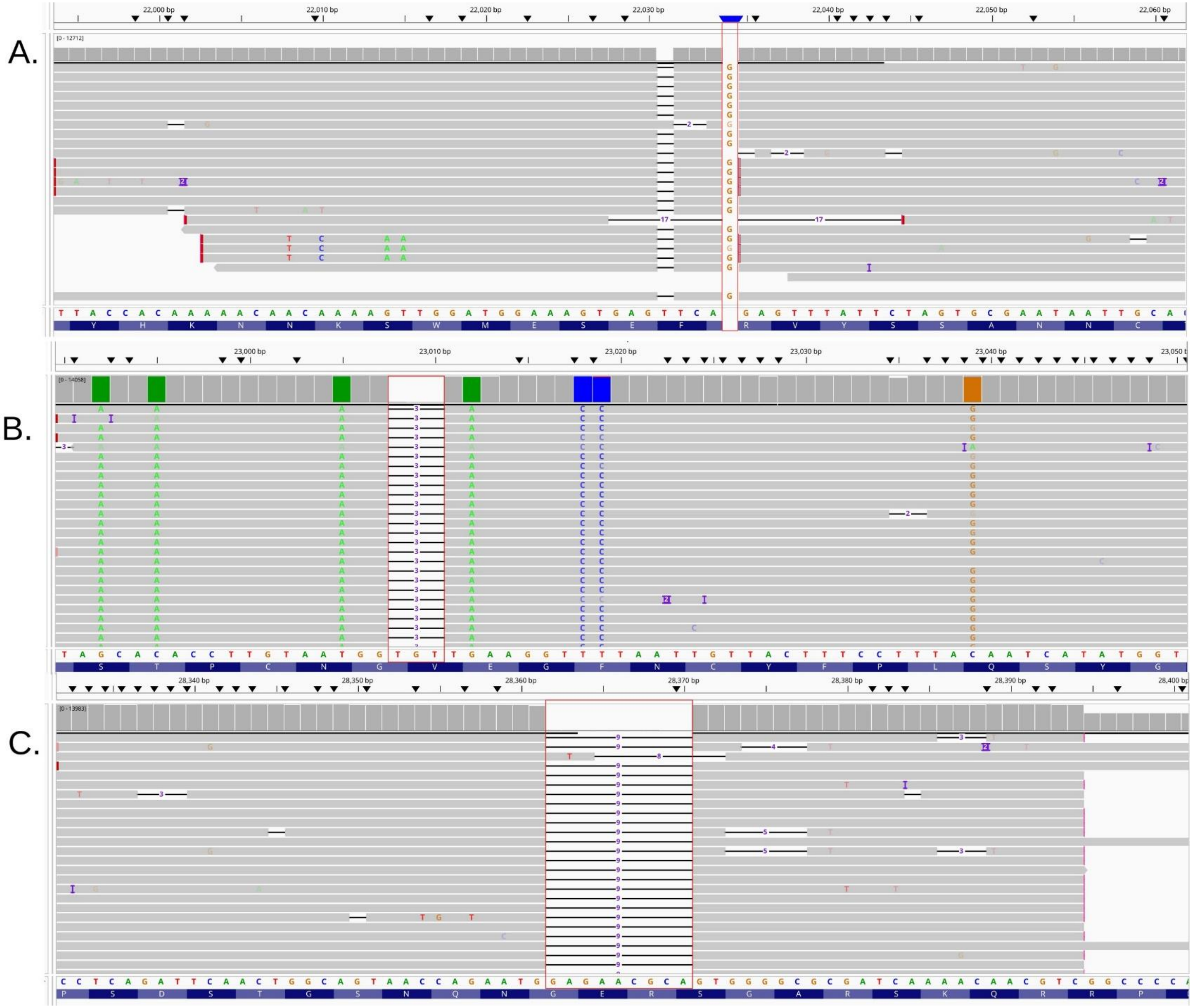

Supplementary Figure S4: Visualisations of refmap obtained reads alignment to the reference sequence; **A.** ins_A22034+G locus within the S gene of SARS-CoV-2; **B.** del_23008- locus within the S gene of SARS-CoV-2; **C.** del_G28368-CA locus within the N gene of SARS-CoV-2.

S.5 Methods

S.5.1 SARS-CoV-2 lineage monitoring program protocol

Nucleic acids were isolated using a Maelstrom 9600 (TANbead inc., Taiwan) instrument loaded with a TANBead OptiPure Viral Auto Plate Kit (TANbead inc., Taiwan) from 300 µL of nasopharyngeal swabs collected in VTM (Liofilchem, Italy) according to the manufacturer's 665-rapid protocol.
The isolates were used for the synthesis of PCR amplicons according to the Illumina COVIDSeq (Illumina, USA) assay protocol with SARS-CoV-2 version 4.1 primers. After the cDNA amplification step, the PCR products were purified using AMPure XP (Beckman Coulter, USA) magnetic beads according to the manufacturer's instructions. The purified amplicons were quantified using the Qubit dsDNA High Sensitivity Assay on a Qubit 3.0 Fluorometer (Thermo Fisher Scientific, USA) and used for NGS library preparation. Libraries were prepared according to the protocol for amplicon ligation sequencing using a Native Barcoding Kit 96 V14 (Oxford Nanopore Technologies, UK). Concentrations were determined using the Qubit dsDNA High Sensitivity Assay on a Qubit 3.0 Fluorometer (Thermo Fisher Scientific, USA) and adjusted to a loading concentration of 100 fmol. Sequencing was performed using MinION R10.4.1 or PromethION R10.4.1 flow cells (Oxford Nanopore Technologies, UK) connected to a GridION or P2 Solo instrument (Oxford Nanopore Technologies, UK). The duration of sequencing was variable (12h – 48h) and depended on the number of active pores.
NGS data was analysed by an in-house developed pipeline called "refmap". The pipeline consists of raw data quality trimming and removing adapters with fastp (Chen, 2023), mapping the trimmed reads to the reference genome SARS-CoV-2 (NC_045512.2) with Minimap2 (Li, 2018) and subsequent primer trimming, variants calling and consensus sequence generation with iVar. Finally, the mapping statistics are generated with samtools (Danecek *et al.*, 2021). This pipeline with all detailed settings is publicly available at https://github.com/NGS-bioinf/refmap/tree/main.

S.5.2 Targeted Sanger sequencing

The nucleic acid isolate stored at -30 °C from a randomly selected pilot sample with mutations of interest (sample 8546) was selected for Sanger sequencing. Suitable primer pairs from the ARTIC network (https://artic.network/) nCoV-2019 sequencing protocol flanking the three regions of interest were selected (Supplementary Table S1).
PCR amplification was performed using PrimeScrtipt™ One Step RT-PCR Kit Ver.2 (TaKaRa Bio, Japan) and the selected primers at final concentration of 0.5 µM. PCR cycling conditions were set as follows: 30 min at 50°C, 2 min at 94°C, 40 cycles of 15 sec at 94°C, 30 sec at 56°C (S-protein gene mutations) or 59°C (N-protein gene mutation) and 1 min at 72°C, followed by a final elongation step of 5 min at 72°C. PCR products were purified using Exonuclease I (Exo-I) and FastAP™ - Thermosensitive Alkaline Phosphatase (both Thermo Fisher Scientific, USA) and used as template for BigDye™ Terminator v3.1 Cycle Sequencing Kit (Thermo Fisher Scientific) sequencing reactions with the appropriate forward or reverse primer (as for amplicon synthesis) according to the manufacturer's protocol. The final purification of the sequencing reaction was performed using a BigDye XTerminator™ Purification Kit (Thermo Fisher Scientific). Sequencing was performed in a SeqStudio8 (Thermo Fisher Scientific, USA) using POP7 polymer and a 50 cm array.
Sanger sequencing data was analysed using CLC Main Workbench Version 7.9.1 (Qiagen, Denmark).

S.5.3 Additional bioinformatic analysis

As a comparative alternative to "refmap", the ARTIC SARS-CoV-2 (wf-artic) with default parameters, located at https://github.com/epi2me-labs/wf-artic was used to analyse the same NGS raw data.
Visualisation of the alignment of reads from refmap to the reference sequence was done using the Integrative Genomics Viewer (IGV) tool (Robinson *et al.* 2011). Consensus sequences from all three sources were further compared by alignment with reference sequences of the Wuhan-HU-1 (NCBI Acc. No: NC_045512.2) genome using MEGA: Molecular Evolutionary Genetics Analysis Version 10.1.7 (Kumar *et al.*, 2024).

S.5.4 Re-analysis of all questionable sequences raw data

Raw data from a total of 197 sequences, uploaded to GISAID from July to September 2024, all containing the annotations ins22036A, A22036G, del23009_23012 and, del28368_28369 was re-analysed using the wf-artic pipeline (https://github.com/epi2me-labs/wf-artic).

S.6 Supplementary tables

Supplementary Table S1: Selected primers (v.4.1) from the ARTIC network nCoV-2019 scheme flanking the regions of interest.

Genomic region of interest	Forward primer	Reverse primer	Amplicon size
S-protein gene: ins_A22034+G	nCoV-2019_72_LEFT	nCoV-2019_73_RIGHT	666 bp
S-protein; gene: del_23012-	nCoV-2019_75_LEFT	nCoV-2019_76_RIGHT	676 bp
N-protein gene: del_G28368-CA	nCoV-2019_93_LEFT	nCoV-2019_94_RIGHT	675 bp

References

Chen,S. (2023) Ultrafast one-pass FASTQ data preprocessing, quality control, and deduplication using fastp. *iMeta*, **2**, e107.

Danecek,P. *et al.* (2021) Twelve years of SAMtools and BCFtools. *GigaScience*, **10**, giab008.

Kumar,S. *et al.* (2024) MEGA12: Molecular Evolutionary Genetic Analysis Version 12 for Adaptive and Green Computing. *Mol. Biol. Evol.*, **41**, msae263.

Li,H. (2018) Minimap2: pairwise alignment for nucleotide sequences. *Bioinformatics*, **34**, 3094–3100.

Robinson, J.T. *et al.* (2011) Integrative Genomics Viewer. *Nature Biotechnology* **29**, 24–26.
